# The 2015 landslide and tsunami in Taan Fiord, Alaska

**DOI:** 10.1038/s41598-018-30475-w

**Published:** 2018-09-06

**Authors:** Bretwood Higman, Dan H. Shugar, Colin P. Stark, Göran Ekström, Michele N. Koppes, Patrick Lynett, Anja Dufresne, Peter J. Haeussler, Marten Geertsema, Sean Gulick, Andrew Mattox, Jeremy G. Venditti, Maureen A. L. Walton, Naoma McCall, Erin Mckittrick, Breanyn MacInnes, Eric L. Bilderback, Hui Tang, Michael J. Willis, Bruce Richmond, Robert S. Reece, Chris Larsen, Bjorn Olson, James Capra, Aykut Ayca, Colin Bloom, Haley Williams, Doug Bonno, Robert Weiss, Adam Keen, Vassilios Skanavis, Michael Loso

**Affiliations:** 1 Ground Truth Trekking, Seldovia, AK USA; 2 Water, Sediment, Hazards, and Earth-surface Dynamics (WaterSHED) Lab, School of Interdisciplinary Arts & Sciences, University of Washington Tacoma, Tacoma, WA USA; 3 Lamont-Doherty Earth Observatory, Columbia University, Palisades, NY USA; 4 Geography Dept., University of British Columbia, Vancouver, BC Canada; 5 Tsunami Research Center, Department of Civil and Environmental Engineering, University of Southern California, Los Angeles, CA USA; 6 Engineering Geology and Hydrogeology, RWTH-Aachen University, Aachen, Germany; 7 U.S. Geological Survey, Anchorage, AK USA; 8 British Columbia Ministry of Forests, Lands and Natural Resource Operations, Prince George, BC Canada; 9 Institute for Geophysics, Jackson School of Geosciences, University of Texas at Austin, Austin, Texas USA; 10 Simon Fraser University, Burnaby, BC Canada; 11 U.S. Geological Survey, Santa Cruz, CA USA; 12 Central Washington University Dept. of Geological Sciences, Ellensburg, WA USA; 13 National Park Service, Geologic Resources Division, Denver, CO USA; 14 Virginia Tech Department of Geosciences, Blacksburg, VA USA; 15 CIRES, University of Colorado, Boulder, CO USA; 16 Texas A&M University Department of Geology and Geophysics, College Station, TX USA; 17 The University of Alaska Fairbanks - Glaciology, Geophysical Institute, Fairbanks, AK USA; 18 National Park Service, Wrangell-St. Elias National Park and Preserve, Yakutat, AK USA; 19 National Park Service, Wrangell-St. Elias National Park and Preserve, Copper Center, AK USA

## Abstract

Glacial retreat in recent decades has exposed unstable slopes and allowed deep water to extend beneath some of those slopes. Slope failure at the terminus of Tyndall Glacier on 17 October 2015 sent 180 million tons of rock into Taan Fiord, Alaska. The resulting tsunami reached elevations as high as 193 m, one of the highest tsunami runups ever documented worldwide. Precursory deformation began decades before failure, and the event left a distinct sedimentary record, showing that geologic evidence can help understand past occurrences of similar events, and might provide forewarning. The event was detected within hours through automated seismological techniques, which also estimated the mass and direction of the slide - all of which were later confirmed by remote sensing. Our field observations provide a benchmark for modeling landslide and tsunami hazards. Inverse and forward modeling can provide the framework of a detailed understanding of the geologic and hazards implications of similar events. Our results call attention to an indirect effect of climate change that is increasing the frequency and magnitude of natural hazards near glaciated mountains.

## Introduction

Climate change is driving worldwide glacial retreat and thinning^1^ that can expose unstable hillslopes. The removal of glacial ice supporting steep slopes combined with the thawing of permafrost in alpine regions^2^ increases the likelihood of landslides^3–6^. Glaciers undercut slopes, priming them for failure by deepening and widening valley bottoms, and by producing steeper valley walls^7^. Additionally, ice loading produces stress fractures in the underlying bedrock, further preparing slopes for failure^8^. As climate warms and glaciers shrink and retreat, they can no longer support rock slopes, and fractures expand as stresses are released. This slope conditioning leads to rock falls, deep-seated gravitational slope deformation, and occasionally catastrophic rock avalanches^4,9,10^.

A further effect of glacial retreat is the creation or extension of bodies of deep water, fresh or marine^11,12^, where tsunamis can be generated efficiently (Table 1). Along the glacially sculpted coastlines of Alaska, Patagonia, Norway, and Greenland, communities, tourism, and infrastructure are becoming increasingly exposed to such landslides and the tsunamis they may generate. Tsunamis in lakes can create flood risk downstream by flowing into inhabited downstream valleys (e.g.^13–15^).

**Table 1:** Tsunamis with runup of 50 m or greater in the past century.

Year	Location	Water body	Cause	Latitude	Longitude	Max runup (m)
1958	Lituya Bay, Alaska, USA	Fjord	Subaerial landslide	58.672	−137.526	524
1980	Spirit Lake, WA, USA	Lake	Volcanic landslide	46.273	−122.135	250
1963	Casso, Italy	Reservoir	Subaerial landslide	46.272	12.331	235
2015	**Taan Fiord, Alaska, USA**	**Fjord**	**Subaerial landslide**	**60.2**	**−141.1**	**193**
1936	Lituya Bay, Alaska, USA	Fjord	Subaerial landslide	58.64	−137.57	149
2017	Nuugaatsiaq, Greenland	Fjord	Subaerial landslide	71.8	−52.5	90
1936	Nesodden, Norway	Fjord	Subaerial landslide	61.87	6.851	74
1964	Cliff Mine, Alaska, USA	Fjord	Delta-front failure	61.125	−146.5	67
1934	Tafjord, Norway	Fjord	Subaerial landslide	62.27	7.39	62
1965	Lago Cabrera, Chile	Lake	Subaerial landslide	−41.8666	−72.4635	60
1967	Grewingk Lake, Alaska, USA	Lake	Subaerial landslide	59.6	−151.1	60
1946	Mt. Colonel Foster, BC, Canada	Lake	Subaerial landslide	49.758	−125.85	51
2004	Labuhan, Indonesia	Open coast	Earthquake displacement	5.429	95.234	51
2000	Paatuut, Greenland	Fjord	Subaerial landslide	70.25	−52.75	50

10 out of 14 tsunamis resulted from subaerial landslides into fjords or lakes in glaciated mountains. Other cases have diverse causes: volcanic eruption (1980), landslide into artificial reservoir (1963), subaqueous delta failure (1964), and earthquake displacement (2004). (Data modified from^53^).

Tsunamis triggered by landslide impact can have an order of magnitude shorter periods and higher runups than those driven by tectonics that have dominated tsunami hazard research in recent years^16^. While tectonic tsunamis typically have periods in the tens of minutes and peak runups extending up to around 30 m, the best studied landslide tsunami, which occurred in 1958 in Alaska’s Lituya Bay, had a period of about 76 seconds and peak runup of 524 m^17^. The geologic traces of the Lituya Bay landslide and tsunami have not been documented, providing no analogue for identification of ancient short-period, large-runup tsunamis in the geologic record, be they caused by landslides, volcanoes, or meteor impacts. The only field data available to constrain these reconstructions are the deposits of the 2000 AD landslide-triggered tsunami in Vaigat Strait, West Greenland^18^, and surficial descriptions of deposits from the tsunami in Grewingk Lake in 1967^19^. The event we describe here in Taan Fiord, Alaska provides the best example to date of a well-documented subaerial landslide that generated a tsunami, and of its impacts on a fjord, coupled with detailed examination of its deposits (see Supplementary Fig. online). This study provides crucial insight into landslide-triggered tsunami processes and the various traces of such events.

### The 2015 Taan Fiord landslide

**Figure 1:**
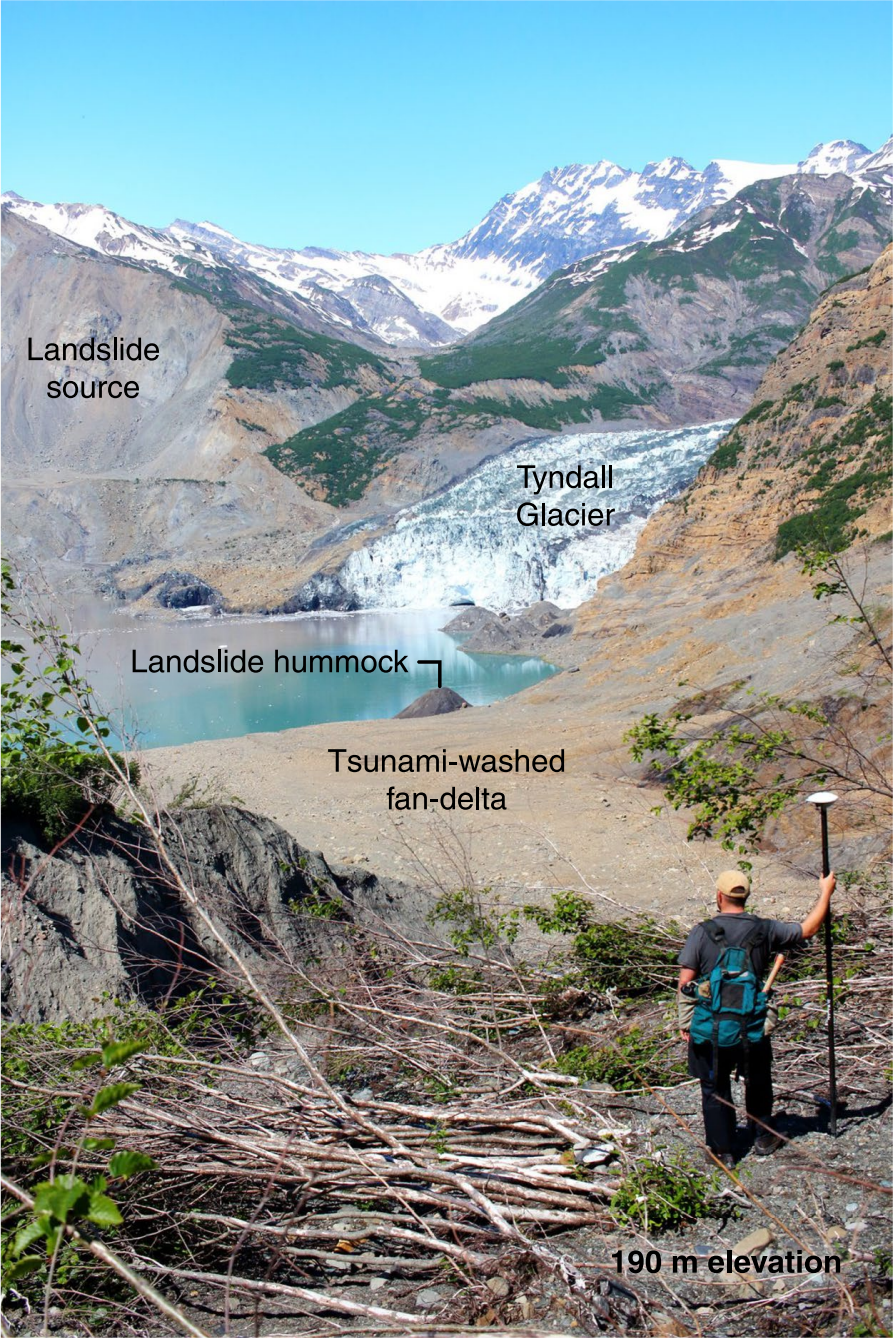
Tsunami impacts near the landslide. The 2015 landslide and tsunami reshaped the landscape at the terminus of Tyndall Glacier. The person in the photo is standing about 190 m above the fjord level, just below the limit of inundation (near the point marked with 193 m runup in Fig. 2).

**Figure 2:**
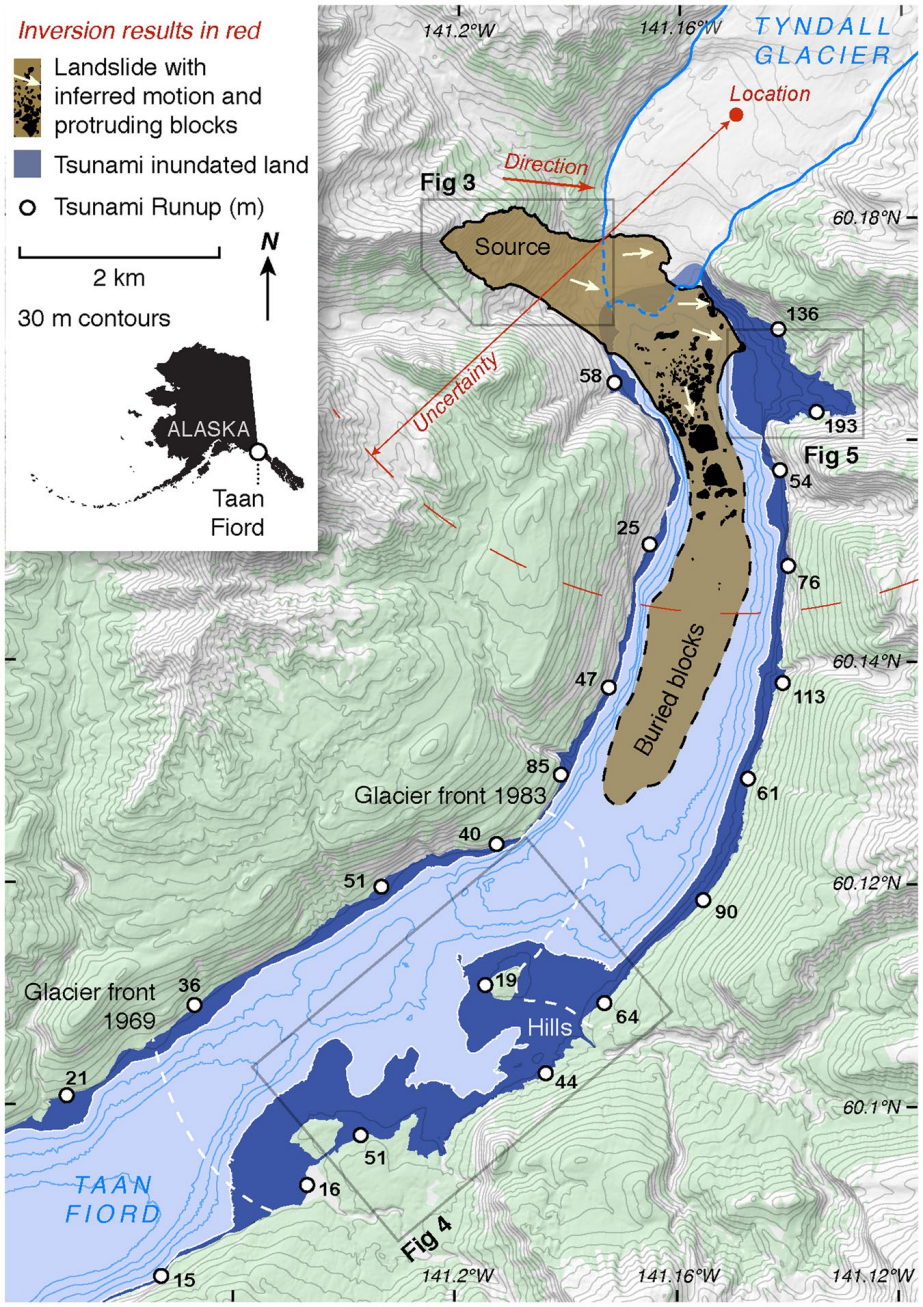
Changes in Taan Fiord. Tyndall Glacier retreated at an increasing pace through the late 20th century until it stabilized in 1991, at approximately the location of the current terminus. The slope failure in October 2015 entered the recently deglaciated fjord at the calving front, generating a tsunami that swept the coast to a height of 193 m. Seismic inversion completed within hours of the event produced an accurate picture of initial motion and a rough location, but could not determine whether the landslide had set off a tsunami. In 2016, marine surveys revealed tens of meters thick blocky submarine runout extending several kilometers^28^. Only the more proximal blocks form submarine hillocks, while more distant ones are buried beneath one or possibly two post-landslide turbidites^28^. Field surveys mapped runup, selected examples of which are presented here. Map created with QGIS 2.18 (http://www.qgis.org/en/site/).

On 17 October 2015, a massive landslide and tsunami occurred at the head of Taan Fiord, an arm of Icy Bay within Wrangell-St. Elias National Park & Preserve in Alaska (Fig. 1). The slope failure was primed by rapid ice loss from a tidewater glacier in a tectonically active setting. Tyndall Glacier filled Taan Fiord as recently as 1961^20^. Rapid warming over the past half century led to 17 km of terminus retreat and over 400 m of ice thinning between 1961 and 1991. Since 1991, the terminus of Tyndall Glacier has stabilized at a shallow bedrock constriction at the head of the fjord^20,21^ (Fig. 2). The slope that failed was above the calving front and slid directly into the fjord along the terminus, partially covering the toe of the glacier. Destruction of vegetation and other tsunami traces clearly delineate runup throughout the fjord. Directly across from the landslide, runup reached 193 m, (as compared to 240 m in an initial model estimate^22^). Runup exceeded 100 m for 1.5 km, overrunning over 1 km^2^ of area. Further down-fjord, Runup varied dramatically, but generally declined to about 15 m at the mouth of the 17 km long fjord (Fig. 2).

Ongoing tectonic deformation likely contributed to the Taan Fiord landslide. The present-day glacier terminus lies along the east-west oriented Chaix Hills Fault, one of many structures that accommodate rapid (4–5 mm a^−1^) tectonic uplift of poorly lithified Miocene-Holocene rocks to high elevations in the St. Elias orogen^21,23^. Uplift of weak and faulted rock likely intensified glacial erosion, leading to rapid valley excavation. Subsequent glacial retreat debuttressed the oversteepened fjord wall, initiating progressive failure of the slope that eventually culminated in catastrophic collapse and a tsunami.

**Figure 3:**
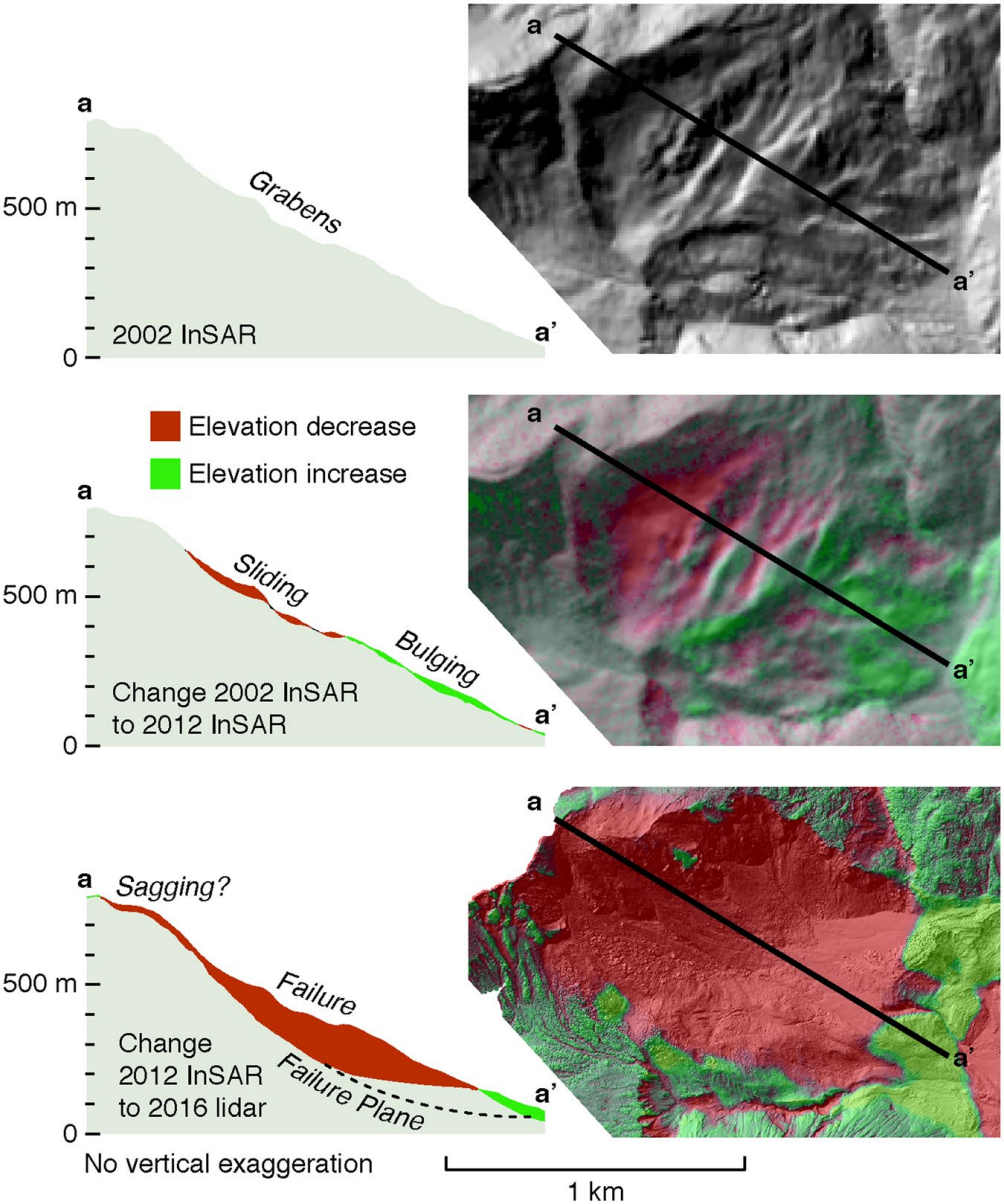
Motion began decades prior to failure. Signs of slope failure in the landslide source area (Fig. 1) were first noted in 1996^19^. Further motion occurred between 2002 and 2012, and the landslide occurred in 2015. Landsat imagery aligned and animated through Google Earth Engine^52^ shows motion progressing yearly during a sequence of images from 1995 to 1998, and that some motion motion (less rapid than 1995 to 1998) occurred between 2010 and 2015. Other portions of the image sequence are too unclear to tell whether motion occurred. The lower panel includes an inferred failure plane from 25. Maps created with QGIS 2.18 (http://www.qgis.org/en/site/).

Signs of prior hillslope deformation at the location of the 2015 landslide might have provided forewarning. Slumping along the fjord wall at the site was first identified in 1996^21^ and grabens are visible in Landsat images as early as 1995. A comparison of Digital Elevation Models (DEMs) and optical satellite imagery show downslope motion throughout much of the ensuing two decades until the catastrophic failure in October 2015 (Fig. 3). While the 2015 Taan Fiord landslide and tsunami did not result in fatalities, actively deforming slopes in more populated places (e.g. Tidal Inlet, Glacier Bay National Park, Alaska^24^) may be harbingers of more deadly landslide-generated tsunamis in the future. Monitoring gradual downslope motion in mountain ranges around the world, while a technical challenge, would provide a step forward in our ability to mitigate risk.

The final trigger for the landslide is unclear. Seismic waves from a M_W_ 4.1 earthquake about 500 km away arrived about 2 minutes before the failure, producing ground motion that would not be uncommon multiple times a year in this area^25^, but might have contributed to the final failure. Similarly, 2015 rains at the nearest gage 110 km away in Yakutat were about 10% more than usual in Sept. and Oct (as-usual the rainiest months of the year). Such deviations above average are common in the years preceding the landslide, but elevated water tables may have contributed at least to the seasonal timing.

### Landslide detection and extent

We first identified the 2015 landslide by seismic inversion, using the method of Ekstrom and Stark^26,27^. The seismic waves of the Taan Fiord landslide, equivalent to a M 4.9 earthquake, were observed globally. We used an automated landslide detector to identify the seismic signal within hours of the event, which included an abundance of 20 to 100 second period energy, as is typical of large landslides^25^. Long-period waveforms from the Alaska Regional Network were used to determine the forces associated with the landslide and to refine the estimated location to within 5 km. Seismic inversion suggested an eastward-moving (bearing 96°) landslide that generated peak forces of about 2 × 10^11^ N and lasting 90 seconds. The landslide source location inferred from seismology was near the calving front of Tyndall Glacier (Fig. 2). Based on earlier mapping of fjord geometry^18^, the seismogenic motion of the landslide was assigned a length of 1.5 km. These findings, combined with the seismologically determined force history, further suggested a slide mass of 1–1.5 × 10^11^ kg. Thus, the Taan Fiord landslide was one of the largest non-volcanic landslides in decades^26,28^.

These initial estimates were revised within the next year by satellite and aerial imagery, lidar, and ground surveys. The landslide above the terminus of Tyndall Glacier unleashed 7.6 × 10^7^ m^3^, or 1.8 × 10^11^ kg of debris. The estimated volume and mass is based on the difference between 2012 and 2016 DEMs, and on an estimate of the slide material remaining in the slide scar. Extending the failure plane beneath onland deposits shows that about 33% of the evacuated volume is still onshore; the rest entered the fjord. Presuming that initial motion was downslope, the landslide moved in a direction similar to that inferred by seismic inversion. The majority of the slide followed the fjord bottom, curving right in an approximately 90° arc blanketing the fjord bottom to its limit 6 km from the source^28^ (Fig. 2). Additional slide material travelled directly eastward through the fjord (Fig. 2) and up onto the far shore, depositing hummocks of semi-coherent slide material that blanket the fjord bottom and crest ~15 m above sea level^29^. This material likely traversed across the bottom of the 90 m-deep fjord and then traveled upwards 105 m to reach its final resting place.

If we assume that the hummocks represent the leading edge of the landslide, the slide velocity must have been at least 45 m s^−1^ (162 km h^−1^) for the Taan landslide, similar to values reported for other rock avalanches of comparable dimensions (1903 Frank slide, Alberta, Canada: 3 × 10^7^ m^3^, 49 m s^−1^; 1912 Mageik, Alaska: 5.4 × 10^7^ m^3^, 24 m s^−1^; 1925 Gros Ventre slide, US-Wyoming: 3.8 × 10^7^ m^3^, 59 m s^−1^)^30^. This estimate is based on the simple conversion of kinetic to potential energy v = (2gh)^0.5^ often used in landslide studies to estimate flow velocity from runup height (h)^31^, assuming no potential energy transfers from the body of the slide to the leading edge. These assumptions can overstate maximum velocities in some cases^31^, but also fail to account for friction or the transfer of momentum to water. Alternatively, the hummocks may represent a later phase of the landslide that travelled over earlier deposits that had partly filled in the fjord. In this case, the hummocks would have traversed water as shallow as 50 m, and the minimum flow velocity for the slide would be closer to 36 m s^−1^ (130 km h^−1^).

### Tsunami generation, propagation, and runup

**Figure 4:**
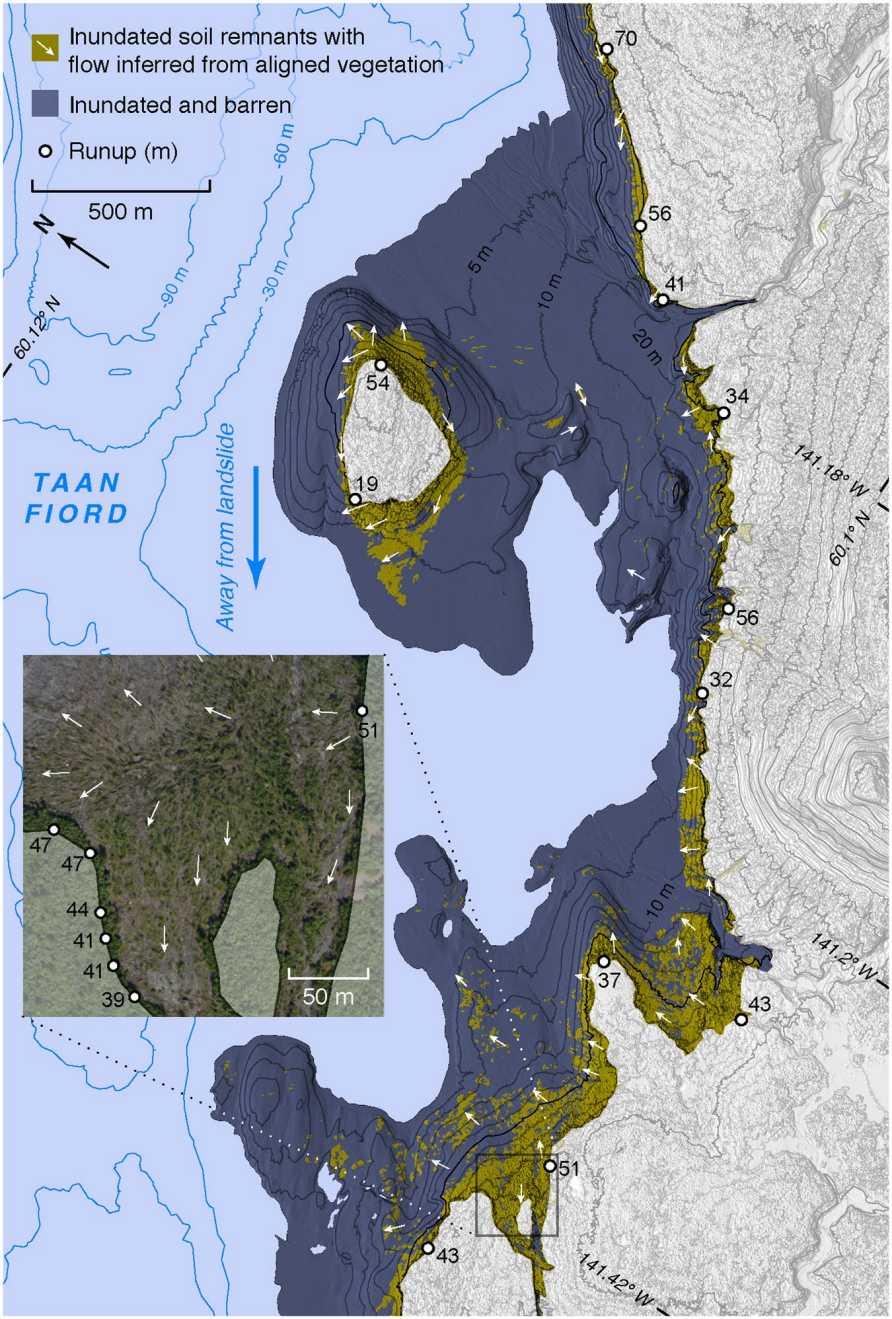
Tsunami recorded by its onshore traces. The Taan Fiord tsunami flooded over 20 km^2^ and left water lines, soil remnants, and flattened, oriented trees. The inset orthorectified imagery includes an example of detailed runup and oriented tree mapping. Bathymetric contours from 28. Map created with QGIS 2.18 (http://www.qgis.org/en/site/).

When landslides enter water, the direct hazard they pose (e.g.^32,33^) can be extended by the resultant tsunami (e.g.^17,18,34^). In Taan Fiord the landslide directly affected about 2 km^2^ of land onshore, while over 20 km^2^ were inundated by the tsunami. We derive the initial tsunami geometry, constrained by landslide volume and aspect ratio, velocity, and duration^35^. Using a coupled set of solid and fluid mechanics models^35^, we estimate that the measured landslide dimensions and material properties generated a leading wave near the head of the fjord with crest elevation of 100 m and period of 90 seconds. In the 100 m water depth near the source area, the front of this wave would have started to break at this crest height, approaching the sloping fan on the far side of the fjord as a plunging or surging breaker. To reach its peak elevation of 193 m (Figs 2, 4), the tsunami required enough initial kinetic and potential energy to not only climb the slope, but also overcome energy lost to turbulent dissipation and sediment interaction.

The tsunami traveled south away from the landslide source area, down the fjord. Shallow water wave theory (aka long water wave theory^36^) computes a propagation speed of (gh)^0.5^, or about 30 m s^−1^ in Taan Fjord’s 100 m deep water. The tsunami proceeded to strip alder forest to elevations exceeding 50 m along the upper 7 km of the fjord, before encountering a range of hills (Fig. 2). These hills caused complex wave interference, expressed as runup elevations that rise and fall by tens of meters across distances of a few hundred meters (Fig. 4). Variability in the flow is further evidenced by uneven stripping of soil, and by the diverse orientations of still-rooted but flattened trees. Farther down the fjord, runup was diminished, reaching between 10 and 30 m elevation. Even with these relatively low runup elevations, the tsunami energy during overland flow was strong enough to leave only soil and debris where young forest with a 10 m canopy previously stood. The leading crest of the tsunami exited the fjord within 12 minutes, based on numerical modeling of the tsunami (see Methods). At distances greater than 5 km from the mouth of Taan Fiord, tsunami runup was below the high tide shoreline and no longer directly measureable during our first field survey six months after the event.

### Geologic traces of the tsunami

The tsunami left thick distinctive deposits that were unlike those documented from other modern tsunamis^16^ as it overran and resurfaced several alluvial fans along Taan Fiord (see Supplementary Fig.). On the hardest-hit fan (Hoof Hill Fan) the change in surface elevation between DEMs from before and after the event showed the deposit exceeded 5 m thick in places. Even at the most distal fan studied, where the tsunami runup had diminished to 16 m, the deposit was still 40 cm thick. These deposits included many fragments of supple wood, sometimes overlaid pre-tsunami soil, and occasionally included uphill flow-direction indicators. Deposits characterized from numerous recent tectonic tsunamis were typically sandy, less than 10 cm thick, and often normally graded^14^. Some of the Taan Fiord tsunami deposits were similarly normally graded as well, however in most ways they were very different. They included abundant coarse sediment ranging up to boulders, and are composed of three distinct units that we could find no analog for in the literature describing tsunami recent historic tsunami deposits.

The three units were most distinct where the tsunami was largest, at Hoof Hill Fan. The lower unit (A) is composed of sand to boulders, while the upper unit (B) is typically well-sorted and composed of cobbles or boulders. A third unit (C), composed of normally graded sand, was found where it infiltrated unit B.

**Figure 5:**
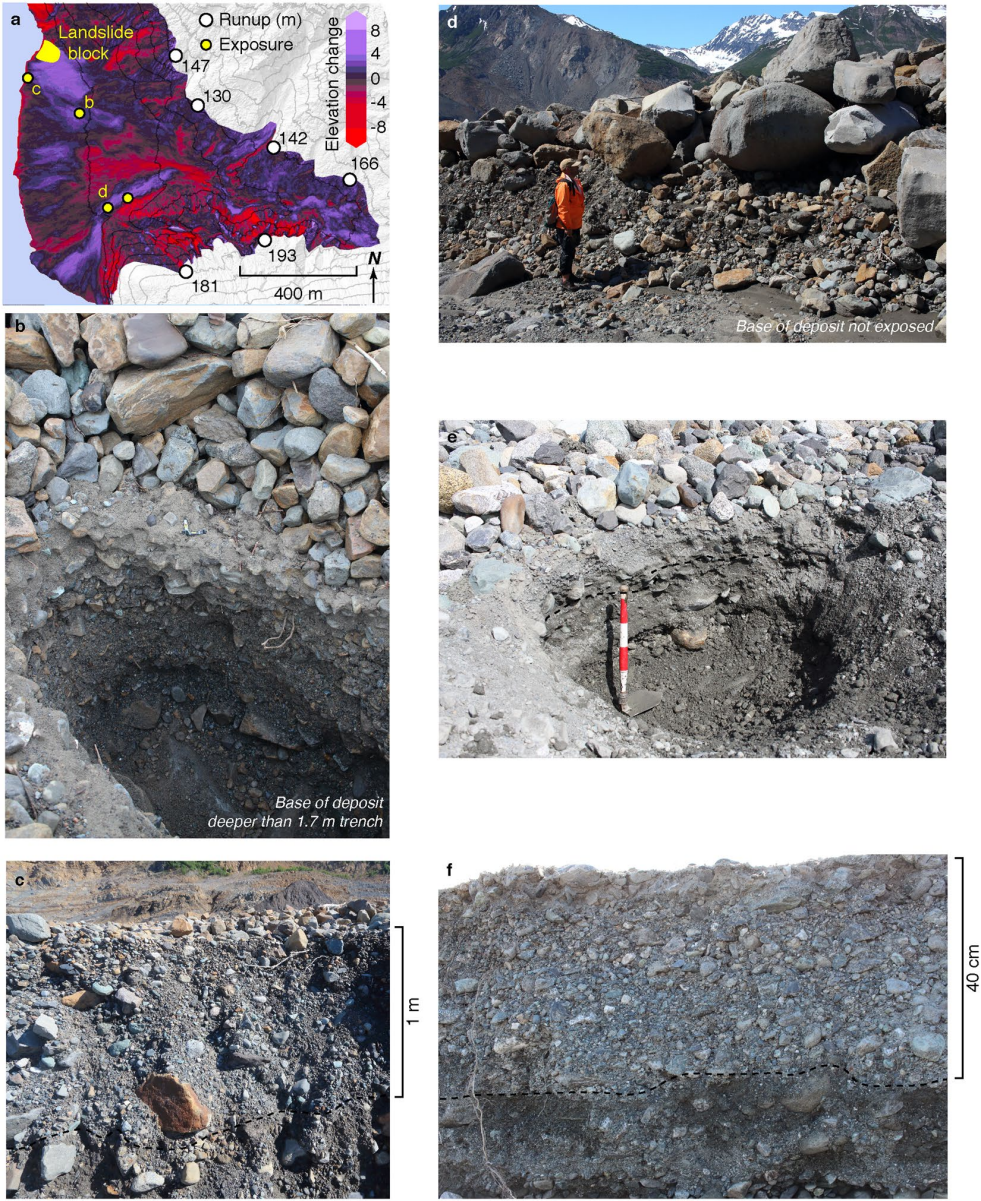
Taan Fiord tsunami deposits. The change in elevation between a 2014 DEM derived from satellite photogrammetry and 2016 lidar data reveals multi-meter changes in surface elevations of an alluvial fan reached by the landslide and swept by the tsunami (**a**). Where exposed in erosional banks or trenches, the deposit included a lower unit of very poorly sorted sand to boulders, and an upper unit of sorted boulders or cobbles (**b**,**c**,**d** - locations noted on map). At the trench in (**b)**, and the outcrop in (**d)**, the pre-tsunami surface was not exposed. However the outcrop in (**c)** extended down below the pre-tsunami surface, exposing siltier, browner sediment (contact dashed). Laterally, portions of the original soil was intact, and included shrubs folded uphill in the direction of tsunami inflood. Further down fjord, similar deposits were found where runup was about 50 m (**e**, contact dashed). Even where the tsunami had diminished to the point where runup was only 16 m, the deposit was still 40 cm thick and included abundant cobbles (**f**, contact dashed). Map in (**a)** created with QGIS 2.18 (http://www.qgis.org/en/site/).

Similar three-part deposits also partially blanketed fans farther down-fjord, although in many cases unit B was thin or absent, and in a few places the deposit was capped by complex layered sediment that we left uncategorized. Unit A might resemble debris flows from upland sources but can be distinguished by evidence of scour and of uphill flow found at the base. Unit B is similar to, but more tabular and widespread than, sieve deposits found on alluvial fans^37^. DEM differencing shows that these deposits are widespread and commonly meters thick at Hoof Hill Fan (Fig. 5), and thus likely to be preserved for millennia. Deposits in more sediment-poor settings are thin and patchy, but include transported boulders up to 5 m in diameter.

The difference between the deposits in Taan Fiord and those that have been recently described in other tsunamis might be due to differences in sediment source, depositional setting, or wave shape, among other things. The difference in deposit composition may simply reflect a difference in sediment source: In both Taan Fiord and tsunami deposits elsewhere, the bulk composition of the deposit is similar to the source sediment, whether that source is sandy beaches or bouldery alluvial fans. Also, a fan is importantly different from a coastal plain because the retreating wave may have the capacity to rework significant sediment as it runs down the sloping surface. In contrast, coastal plains drain more slowly, and typically sediment is only mobilized in localized constrictions during withdrawal^16^. Finally, while tectonic tsunamis have a long period, usually over 10 minutes, the period of the Taan Fiord tsunami was likely similar to the 90 seconds it took the slide to do most of its acceleration and deceleration. This difference in period likely had large impacts on temporal and spatial variability in the tsunami flow as it moved onland, and thus on the erosion and deposition of sediment.

The tsunami deposits in Taan Fiord may be particularly useful in identifying or interpreting deposits of similar events that produce short-period waves, and send those waves over sloping surfaces with diverse sediment available. For example, deposits interpreted to have been generated by landslide tsunamis have been documented in Hawaii^38^ and the Canary Islands^39^, pre-Quaternary deposits in the rock-record have been interpreted as records of large landslide or impact-generated tsunamis (summary in 14), and possible impact-generated tsunami traces have been described on Mars^40,41^. The deposits in Taan Fiord provide the first well-constrained example that might be used to interpret these and other ancient deposits, in order to better understand the frequency and magnitude of landslide and bolide impact tsunamis. If viewed independently, and out of geomorphic context, neither of the sedimentary units left by the Taan tsunami are necessarily indicative of a landslide-triggered, short-period tsunami. However, taken together and contextualized with the other evidence, the sedimentary deposits may prove sufficiently distinct to aid in the identification of paleo tsunamis. We provide more detailed descriptions of the tsunami sedimentology in the Supplementary Figure.

### Implications for hazards assessment

The landslide and tsunami predicated by glacial retreat at Taan Fiord represents a hazard occasioned by climate change. More such landslides are likely to occur as mountain glaciers continue to shrink and alpine permafrost thaws. These landslides can more often be expected to produce tsunamis as water bodies grow and extend landward, closer to steep mountain slopes. Other notable landslides have occurred in recently deglaciated regions (e.g.^3,4,9^) and some have produced tsunamis (e.g.^17,19,34,42,43^, Table 1). Their locations, though mostly remote, are attracting tourism and development. For example, incomplete failure and ongoing slow slip have been documented on a slope at Tidal Inlet, a fjord in Glacier Bay National Park, 6 km from a channel visited by dozens of cruise ships during summer months^24^. On 28 June, 2016, an approximately 1.5 × 10^11^ kg landslide collapsed onto Lamplugh Glacier, also in Glacier Bay National Park, but luckily did not reach tidewater and so did not trigger a tsunami^44,45^. Then on 17 June, 2017, a landslide in Rink Fiord triggered a tsunami that killed 4 people in Nuugaatsiaq, Greenland, 30 km away, highlighting the need for further study of these phenomena.

Comparison of pre- and post-event data at Taan Fiord constrain the dimensions of the landslide and tsunami, and in turn may aid in identifying other such events in the recent geologic past. In order to mitigate the risk associated with landslide-triggered tsunamis, we suggest the following: 1) revisit geologic records of paleotsunamis to better understand frequency and causal mechanisms of past occurrences; 2) assess areas of potential failure given known glacial histories and evidence of precursory motion; and in areas of particular concern, 3) map areas of likely impact using glacier, landslide, and tsunami inundation models in order to reduce impacts should an event occur; and finally 4) monitor for landslides using seismic and remote-sensing techniques.

## Methods

### Remote-sensed topography

For our landslide volume estimate, documentation of precursory motion, and mapping of deposit thickness (Fig. 5) we used a variety of Digital Elevation Model (DEM) and imagery sources. The earliest DEMs are taken from airborne interferometric synthetic aperture radar data (InSAR), while more recent DEMs come from satellite photogrammetry, as well as airborne lidar and structure-from-motion (SfM). The InSAR-based DEM’s are taken from 2002 and 2012; these are freely available 5-m resolution models, and can be downloaded at http://maps.dggs.alaska.gov/elevationdata/#-16000000:9338001:4. We construct satellite DEMs, including the 2014 DEM referenced in Fig. 5, from high-resolution along-track stereo optical satellite imagery using SETSM^46^ on the University of North Carolina Chapel Hills’ Killdevil HPC system. The resulting DEMs have a resolution of 2 m. The optical DEMs are co-registered to the 2012 InSAR DEMs by masking out all water and snow and ice from the InSAR DEMs, leaving only bedrock, converting the InSAR bedrock into a pointcloud then applying an iterative closest point matching routine to minimize the RMS difference between the optical DEM and the InSAR DEM. DEMs used in this study are available from both the Polar Geospatial Center at the University of Minnessota, and similar DEMs of the area, registered to satellite altimetry using different methods than this study are available from the ArcticDEM project (Arcticdem.org).

During 2016 fieldwork we collected lidar and imagery for SfM over Taan fjord and parts of Icy bay. The lidar was collected using methodology from^47^, and SfM topography and ortho-imagery was produced using methodology in^48^.

DEM differencing shows that 5.8 × 10^7^m^3^ of landslide material is missing - providing a minimum volume on the landslide. By extrapolating the failure plain beneath slide material still onshore, we expand this estimate to 7.6 × 10^7^m^3^. To estimate the mass represented by this volume, we used a density of 2350 kg/m^3^. Most of the landslide material is a weakly lithified sandstone. Sandstones have density values between 2150 and 2650 kg/m^3^ ^49^. The value of 2350 kg/m^3^ is based on rocks from the Susitna and Cook Inlet basins, Alaska, which have a similar composition, age, and burial history (e.g.^50,51^).

To understand precursory motion, we compared successive DEMs, and also reviewed imagery provided through Google Timelapse^44^, which presents Landsat imagery that is clear enough for identifying landslide scarps and grabens back to 1995.

### Runup survey

We used a variety of instruments to perform the ground-based tsunami runup survey. The instruments that were used to survey the tsunami markers included a laser rangefinder, a total station, and two differential Real Time Kinematic (RTK) GPS systems. A combination of these instruments was commonly used for a single measurement point, as the terrain was difficult and flow markers were at times inaccessible. There were four different general approaches for surveying data points in this survey. The first approach was used for markers located on steep mountain slopes, where run-up was clear, but not easily accessible, and required shooting from a distance. For such points, the RTK-GPS system was used in combination with the rangefinder to obtain a spatial location. A second approach was employed in situations where the ample forestry or tree tops obstructed the RTK-GPS signal. In this scenario, a combination of the total station and the RTK-GPS system was used to obtain the necessary coordinate points with high accuracy. Thirdly, for flow markers of large elevation (e.g. more than 10 meters) and/or large inundation distance (e.g. many 100 s of meters) that were also entirely accessible due to relatively easy terrain and lack of dense canopy, the RTK-GPS system was used on its own. Finally, for flow markers of relatively low elevation and inundation distance, the rangefinder on its own was used in locations where it was not practical to setup the RTK base and rover system. In the paragraphs below, we briefly describe each of the measurement devices, and their expected precision.

The RTK-GPS system was used to in order to achieve highest possible accuracy. Survey monuments were created throughout Taan Fiord, and their coordinates were determined via static GPS measurements. Many monuments were established to accommodate the radius of radio coverage of the RTK-GPS system. The expected errors are +/−1 cm for the horizontal, +/−3 cm for the vertical measurement using RTK-GPS. In addition to instrument error, some human error must be taken into account. The human error is estimated to be +/−5 cm in holding the pole upright and on stable ground. Thus the RTK-GPS systems are expected to have measurement errors bounded by 10 cm.

When using the total station, the instrument location was determined by acquiring its relative location to four well-distributed points with known coordinates. Following the setup of the total station, a surveyor used a reflector to collect the tsunami markers. The errors for a typical distance of ~200–300 m are +/−1.5 mm for the distance, +/−0.001° for the inclination, and +/−0.001° for the bearing. The human error was again determined to be +/−5 cm in holding the pole upright and on stable ground.

When used in conjunction with a RTK system or the total station, the rangefinder location was determined from the RTK rover, and the distance/vertical angle/bearing measurements from the rangefinder provided the coordinates of the tsunami marker. The errors for a typical distance of ~50–10 0 m are +/−30 cm for the distance, +/−0.25° for the inclination, +/−1° for the bearing. The human error in holding the rangefinder vertically and steady was determined to be +/−10 cm.

As discussed above, the expected errors of each measurement are strongly dependent on the equipment used as well as the local terrain, but are likely less than 10 cm for measurements not using the rangefinder, and less than 30 cm for measurements using the rangefinder. In addition, the tsunami marker elevations need to be presented as relative to the tidal level at the time of the tsunami. Of course, there are no direct measurements of the sea level in Icy Bay during the tsunami. Based on sea level data measurement during the field campaigns, we find that the tides within Icy Bay match those at the NOAA tide station in Yakutat, to within 14 cm at high and low tide, with a mean RMS error of 3 cm. Therefore, the error associated with referencing the runup measurements to the sea level at the time of the tsunami is on the same order or smaller than the error in the runup measurements themselves.

### Tsunami source model

To approximate the landslide motion and initial generation of the tsunami, we use the 3D Simplified Arbitrary Lagrangian Eulerian model (iSALE) in the area within 3 km of the slide. The slide geometry and motion are constrained by observations of the failure area, and the force time-history of the slide. We use the tsunami generated by iSALE to specify the initial condition in the weakly dispersive, nonlinear Cornell University Long and Intermediate Wave Model (COULWAVE). COULWAVE simulates the tsunami evolution through Taan Fiord and Icy Bay, including relevant dissipation from wave breaking and bottom friction (results not presented in this paper). Ref.^30^ contains technical details on both of these models, as well as details of the coupling.

### Tsunami Deposits

To characterize the Taan tsunami deposits, we documented outcrops and trenches across several alluvial fan-deltas. Outcrops were opportunistic, while most trenches were chosen to be in areas of relatively little variation in the surface of the deposit. Trenching and documenting was extended down to the upper part of pre-tsunami sediment wherever possible. Each was described, photographed, and sampled, with particular attention being paid to grain-size variability and contacts within the tsunami deposit. We provide descriptions of the outcrops in the Supplemental Figure.

Tsunami deposits were easily distinguished from underlying pre-tsunami alluvial fan-delta deposits. We documented tsunami deposits within 9 months of the tsunami, and fragmented wood that had been living at the time of the event remained supple and green where it was trapped in the deposit. These provided clear markers for tsunami deposits. In some cases, pre-tsunami soil, roots, and rooted vegetation highlighted the base of tsunami deposits, though often the soil was scoured away, and the decades-old soil was typically faint.

### Marine Geophysical Surveys

During Summer 2016, we acquired multibeam bathymetry data from the R/V *Alaskan Gyre* and from a remotely operated surface vehicle the *Jokull* for all of Taan Fiord. Also, multichannel 2D seismic data were acquired from the *Alaskan Gyre*. These data are discussed explicitly in 28 and 29 and are only used here to provide a general description of the submarine part of the landslide.

## Electronic supplementary material

Supplementary Figure

## Data Availability

Grain size distribution data available upon request to hig314@gmail.com.
