# Supplementary material for: The 2015 landslide and tsunami in Taan Fiord, Alaska
Source: Sci Rep. 2018 Sep 6;8:12993. doi: 10.1038/s41598-018-30475-w (PMC6127189; doi:10.1038/s41598-018-30475-w)
Supplement: Supplementary file 1 — Supplementary Figure [file 41598_2018_30475_MOESM1_ESM.pdf]

Supplementary Figure for: The 2015 landslide and tsunami in Taan Fiord, Alaska

Authors: Bretwood Higman, Dan H. Shugar, Colin P. Stark, Göran Ekström, Michele N. Koppes, Patrick Lynett, Anja Dufresne, Peter J. Haeussler, Marten Geertsema, Sean Gulick, Andrew Mattox, Jeremy G. Venditti, Maureen A. L. Walton, Naoma McCall, Erin Mckittrick, Breanyn MacInnes, Eric L. Bilderback, Hui Tang, Michael J. Willis, Bruce Richmond, Robert S. Reece, Chris Larsen, Bjorn Olson, James Capra, Aykut Ayca, Colin Bloom, Haley Williams, Doug Bonno, Robert Weiss, Adam Keen, Vassilios Skanavis, Michael Loso

TAAN TSUNAMI DEPOSIT VARIABILITY

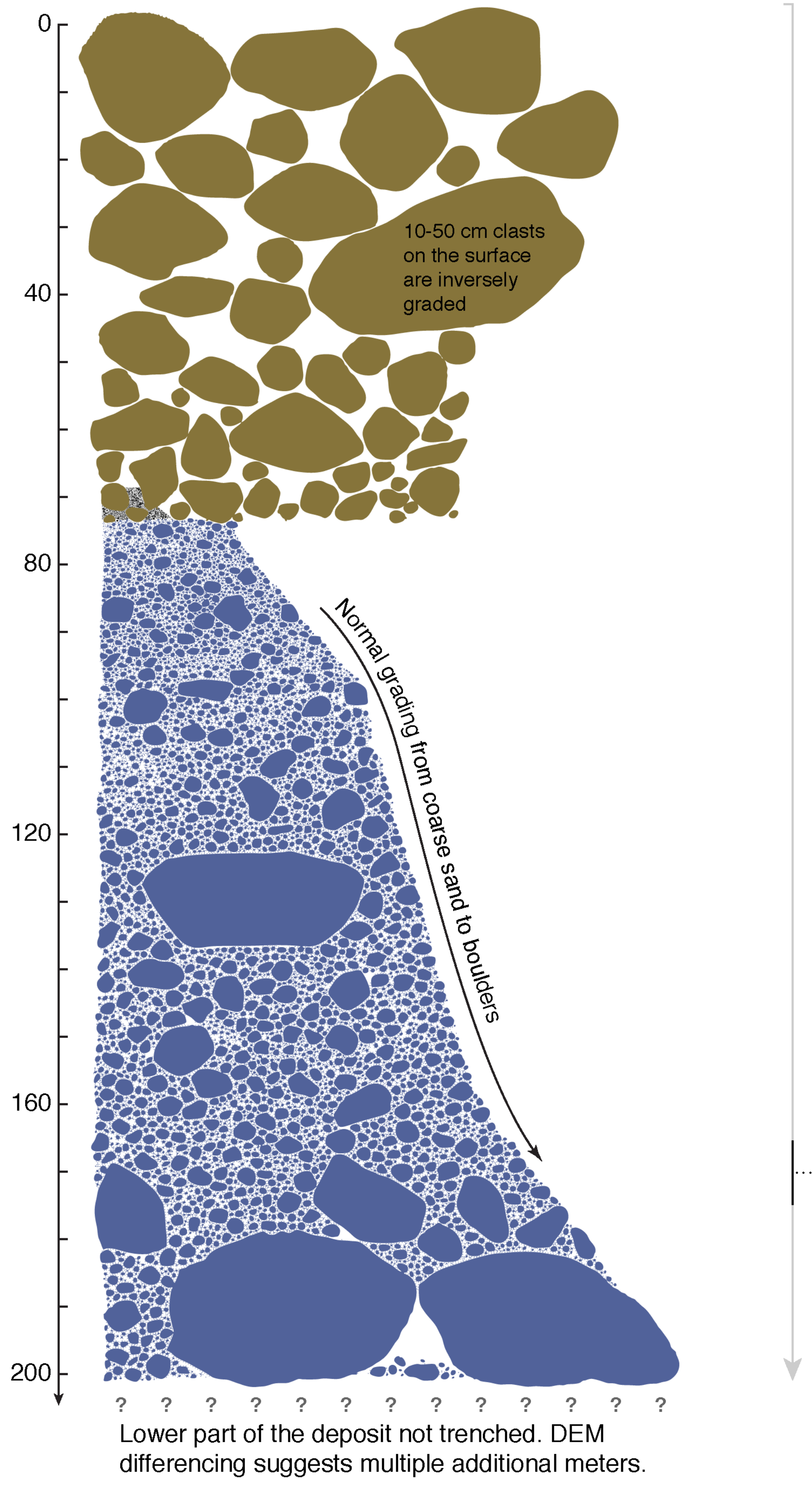

Clasts and sections 1/10 scale

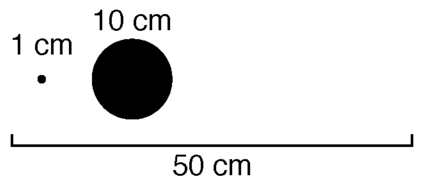

Grading diagrams of Taan Fiord deposits

The six diagrams below are simplified, scale drawings of tsunami deposits in Taan, representative of about 15 sections that we documented. The vertical scale and grain-size are 1/10 real size. The right edge of each diagram approximates variability in mean grain-size. These grain-size values are measured using a Dynamic Image Particle Size Analyzer (CAMSIZER P4) in cases where the grains include few or no grains larger than 3 cm - the limit for Camsizer analysis. In other cases they are estimated based on photos, sometimes informed by grain-size distributions of the smaller sediment classes. In total, we analyzed 75 samples from unit A, 102 from unit B, none from unit C, 28 from unclassified surficial sediment, and 11 from underlying pre-tsunami deposits. Vertical thickness of samples varied dramatically depending on sampling feasibility - from 0.5 cm to 50 cm. Grain-size analysis results are available upon request.

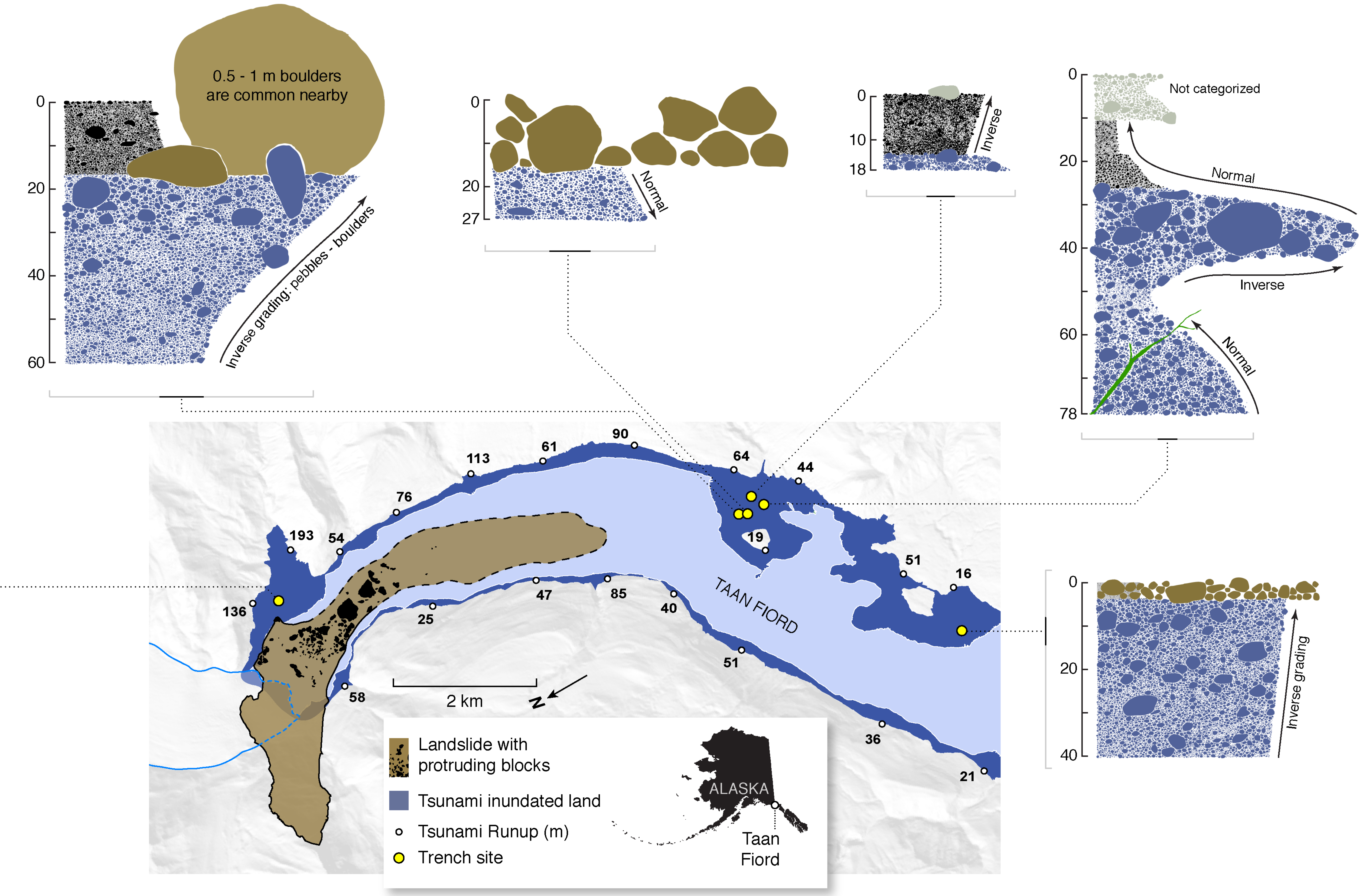

UNITS AND COMPARISON TO OTHER DEPOSITS

		**Characteristics of Taan Fiord tsunami deposits**				**Comparison of Taan Fiord tsunami deposits to tectonic tsunami depostis**[2,3,4,5]		**Comparison of Taan Fiord tsunami deposits to normal alluvial fan deposits**[6]	
		**Grainsize**	**Sorting**	**Grading**	**Other**	**Similarities**	**Differences**	**Similarities**	**Differences**
Unit C	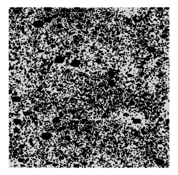	Sand with some pebbles	Moderate to poor sorting (mean graphical standard deviation[1] 1.1 ϕ)	Typically normal	Thickest in saddles along ridges overtopped by tsunami<br>Sometimes fills interstitial space in Unit B	Sandy, normally graded	Thickens in saddles	*Unlike common alluvial fan deposits*	
Unit B	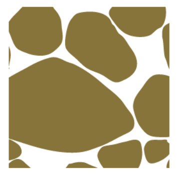	Boulders or cobbles	Not measured, but appears moderately sorted	Inverse where more than one grain thick	Typically hosts large interstitial spaces except where packed with sand from unit C.<br>Often patchy	*No known example for comparison*		Similar grain-size and sorting to sieve deposits	More widespread and continuous than sieve deposits
Unit A	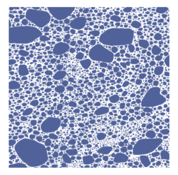	Diverse - sand to boulders	Poor to very poor for < 3 cm grains (graphical standard deviation[1] from 1.25 to 3 ϕ, with a mean of 1.8 ϕ).<br>This analysis likely understates grain diversity because it excludes cobbles and boulders.	Normal or inverse	Flow-direction indicators show deposition during inflood.<br>Trapped voids beneath large clasts.<br>Rare armored mud-balls	Sheet-like<br>Deposit composition mirrors source	Thicker, coarser.<br>Thick deposits extend up slopes as steep as 10%.	Debris flow deposits are also poorly sorted and include coarse sediment	Uphill flow indicators<br>Very little mud<br>Voids beneath large clasts<br>Deposit extends to slopes above the alluvial fan

BIBLIOGRAPHY

1.Folk, R.L. Petrology of sedimentary rocks. *Hemphill Publishing Company*, (1968).

2.Bourgeois, J. Geologic effects and records of tsunamis. *The Sea* **15**, 53–91 (2009).

3.Dawson, A. G., and Shi, S. Tsunami deposits. *Pure and applied geophysics* **157** 6-8, 875-897 (2000).

4.Goto, K., et al.. Spatial thickness variability of the 2011 Tohoku-oki tsunami deposits along the coastline of Sendai Bay. *Marine Geology* **358**, 38-48 (2014).

5.Higman, B. Parsing the sandy onshore deposits of modern tsunamis. *Ph. D. Dissertation, University of Washington, Seattle* (2007). https://digital.lib.washington.edu/researchworks/handle/1773/3232

6.Hooke, R. L. Processes on Arid-Region Alluvial Fans. *The Journal of Geology* **75**, 438-460 (1967).
